# Address of C. B. Boothe, President of the California Association for the Study and Prevention of Tuberculosis, at Annual Meeting, December 3, 1907

**Published:** 1908-04

**Authors:** 


### ADDRESS OF C. B. BOOTHE, PRESIDENT OF THE CALIFORNIA ASSOCIATION FOR THE STUDY AND PREVENTION OF TUBERCULOSIS

AT ANNUAL MEETING, DECEMBER 3, 1907.

When big battleships begin to maneuver, they find it convenient to use a small gun to get the range. My duty here is simply to bring you within range of the real fighting forces in this warfare, and I shall engage but a very few moments of your time, for some of our big guns are here and ready to be aimed at you.

I know of no more appropriate introduction of what I shall have to say in this beautiful edifice, dedicated to God, than to quote from His word certain paragraphs, which indicate that the warfare in which we are engaged for the benefit of humanity, for the protection of our homes, firesides and loved ones, was being waged at the dawn of written history.

We have reason to believe that those nations who have lived largely in the open air and pursued out-of-door occupations, have to a considerable extent, escaped the excessive ravages of this disease, which flourishes in thickly settled communities among those who have indoor occupations, or dwell in unhygienic environment.

While the laws given in that greatest and oldest of law-giving Books, Leviticus, are both religious and ceremonial, they are also civil, moral and sanitary; especial stress being placed upon cleanliness and sanitation.

In the 16th verse of the 26th chapter, God is quoted as speaking to the children of Israel, and saying to those who would not harken to Him and do His commandments—"I will do this unto you; I will appoint over you terror, consumption, and a burning ague, that shall consume the eyes and cause sorrow of heart." And in Deuteronomy, the 22nd verse of the 28th chapter, the blessings of obedience and the curses for disobedience are set forth, and the first and most dreadful curse for disobedience of His laws is stated as follows: "The Lord shall smite thee with a consumption, and with a burning fever, and with inflammation and with an extreme burning, etc."

While Deuteronomy means the second giving of the Law for a generation yet unborn, and covers the period only of the last month spent by the Israelites on the plains of Moab, just before entering Canaan, at the end of the forty years wandering, it is in the 19th verse of the 30th chapter that is found the real secret of the book, in these words: "I call heaven and earth to record this day against you, that I have set before you life and death, blessing and cursing; therefore choose life, that both thou and thy seed may live."

If you will recall that the great leader, Moses, and many of those still with him, had come out of Egypt, and he often refers to pestilence and deadly diseases which were common there, and that he promised exemption from the Egyptian susceptibility to disease if they followed the laws which he set forth for them, it may well be believed that Tuberculosis, or Consumption as it is frequently called, had become a "terror" in the eyes of Moses while he lived in Egypt, for he graphically describes it; and it is believed that this disease did much to depopulate that land; and furthermore, one of our prominent scientific investigators has recently advanced the statement that the tidal wave of this dread disease, which has occurred in various sections of the world, is due to the shipment and exposing of mummified human bodies, incident to the remarkable excavations carried on in Egypt and thereabouts; it being noted that the alarming spread of the disease had been co-incident with the exhibitions of these mummies from the ancient tombs. If there had been any great incursion of tuberculosis during the intervening time, I am unable to say, but during the last generation or two it has gained enough headway to cause scientific men to grapple with it.

England was the first country to recognize the necessity of better hygiene in homes, and the first nation to establish hospitals for those suffering from the many forms of tuberculosis.

In 1863, Parliament enacted a law governing the hygienic construction of houses. This was followed by laws demanding better workshops and sanitary homes for the workers.

A national association for the prevention of tuberculosis was formed in 1899, with the Prince of Wales as President. It has grown steadily up to the present time. In Great Britain there are about seventy establishments for the care of consumptives, the majority being for paying patients. There is still great need there for providing accomodation for the poor consumptives.

About 1896, in Germany, 800 distinguished citizens formed an association, of which the Empress is the Protectress, Chancellor von Bulow, the Honorable President, and General von Pannivitz, the Secretary. This association began the work of erecting what is called "Folks Sanatoria" or "People's Helping Stations," and this work has progressed so earnestly that now there are over 200 of these institutions in full operation, and between 12,000 and 14,000 tuberculous patients are continuously cared for in this way.

In 1899, a Congress was held in Germany to consider the subject of tuberculosis, which served to popularize the prevention of it among the poor.

As early as 1859, Dr. Brehmer, of Silesia, wrote several articles on the sanitorium treatment of tuberculosis. For a long time these articles remained unnoticed, both in Europe and in this country, but came finally to be recognized as the basis upon which the "open air treatment" of this disease is carried out all over the world.

Dr. Brehmer demonstrated by actual experience that "a life spent entirely out of doors in any kind of weather, good and abundant food and rest and discipline," are the chief requisites to utilize to bring about a cure.

Germany undoubtedly has the most numerous institutions for the relief of poor consumptives; they are also the best organized, showing the most definite results of any in Europe. Perhaps it is not too much to say that the world is indebted to Germany, and Germany to Drs. Brehmer and Dettweiler above all others, for the establishment of these sanatoria, Dettweiler having founded the first sanatorium for the poor at Ruppertshain. His sanatorium at Falkenstein is known as the mecca for students of phthisiotherapy from all over the world. Quoting from a tribute recently paid to Dr. Dettweiler—"He was a charitable man of unusual cordiality and kindness, yet strong in personality, stern and severe when occasion demanded it. He studied the whole life of every patient and was friend, confessor and physician."

I may take this opportunity to say that Dr. Dettweiler received his impulse and faith in the sanatorium treatment of consumption from Dr. Brehmer, whose patient he was for several years. Likewise, from Brehmer, came the inspiration to Dr. Trudeau, who, some twenty years ago, established what has grown to be a magnificent work at Saranac Lake on similar lines, and from Dr. Trudeau radiated the inspiration to our own Dr. Barlow, who has laid the foundation of an institution filling a distinct need, which has already been able to confer great benefit upon many indigent consumptives and promises for the future continued splendid work.

France, so far, has not done much for the tuberculous adult in providing sanatoria, but has directed her efforts mainly to the establishment of dispensaries in the midst of her most populous cities with very satisfactory results. There are a few isolated sanatoria in France, but the principal effort seems to have been to combat the disease in childhood.

Austria has also a number of sanatoria, the one best known being at Alland, some sixteen miles from Vienna, but this institution is only available to men in the early stages of the disease.

In Switzerland there have been established many sanatoria, reported to be well organized and showing excellent results.

Berlin can point to nine outdoor camps, six being for consumptive patients, the others for scrofulous cases and anaemic children. Schools are maintained in connection with these camps. The day should be hastened in this State when such outdoor camps are established, as they can not but be of great value, not only for immediate relief, but for the education which is radiated by way of getting a better understanding of health laws and the value of direct sunshine and fresh air.

Before referring to what has been done in our own country, I desire to mention that the Central International Bureau, made up of members from all nations, with headquarters in Berlin, established in 1902, is kept informed through correspondence of what is being done in all parts of the world as to the best methods for the prevention and cure of tuberculosis, and the information distributed from that Bureau is looked upon as authentic and of great value.

Other European countries have done something, but those above noted have reached the stage of the greatest effectiveness.

Up to two years ago, when the National Association held its first annual meeting, there were definite state societies in New Hampshire, Vermont, Pennsylvania, Maryland, Ohio, Indiana and Illinois. During the past two years new organizations have been organized in eight states—New Jersey, Delaware, North Carolina, Georgia, Kentucky, Minnesota, Iowa and Washington. Missouri, Rhode Island, Wisconsin, New York, Virginia, Michigan and California have plans well advanced, some of them practically completed. Several large cities like Boston and New York have each a local society, acting as a stimulating center for work in other communities of the State.

The census of 1900 shows 38 cities in the United States with a population of over 100,000. Up to two years ago, fifteen of these cities had organized societies for the prevention of tuberculosis. During the past year, in 11 additional cities, associations have been organized, and of the remaining 12, 7 have organizations under way, leaving only five unawakened.

You will hear this evening from Dr. Rupert Blue, who will, I hope, tell you something of the sanatoria established by our government.

Time will not permit me to give more than general data on this subject, but it may be broadly stated that every part of the

United States has received an awakening touch and the most important centers have responded energetically. In a large number of smaller communities, organizations have been formed and thousands of people have been aroused to the necessity of active co-operation in stamping out the disease.

The Fifth International Congress on Tuberculosis will be held in Washington next autumn, from September 21st to October 12th. This is the first time this Congress has arranged to meet in this country and it may not come here again in a generation. Official delegates will be present from almost every country in the civilized world, and the interest of the Federal Government has been enlisted. All of the departments of the government, excepting two, the Attorney General's and the Postmaster General's, have signified their intention to participate and have petitioned Congress for the necessary authority and means, while most of the Governors of the states have taken official action. There will be a course of thirty official lectures, which all members of the Congress and the general public are invited to attend; but the main value will be in the expert discussions and the more general intimate comparison of experiences and opinions among those who will constitute the regular membership of the gathering.

In California, through the initiative of Dr. F. M. Pottenger, this Association was organized in 1902. Dr. Pottenger was its first President. He was succeeded by Dr. H. G. Brainerd in 1905, and a year ago a layman was selected with a view of securing the co-operation of the general public, by divesting the League of the appearance of being purely a medical association. Under the auspices of the League, has been carried on an intermittent educational work mainly by Drs. Pottenger, Browning and Kress. During the past year, others have been enlisted in this work, as will appear in the report of the Secretary, which you are to hear later.

The Health Officers of Boards of Health, have, in many localities, identified themselves with the work of the League and given efficient direction and aid to our efforts.

A beginning in remedial legislation has been brought about through the efforts of the League in conjunction with the State Board of Health, in the enactment of a law requiring reporting and registration, not only by the medical profession, but by all to whom knowledge shall come of a case of infectious disease.

This law is coming slowly to be understood and complied with, but copies of so much of the Act as refers to this matter should be printed and distributed in every community in the State. There are some details yet to be worked out before this Act becomes as effective as it should.

In the early summer of 1906, a committee was appointed by the Board of Directors of this Association, to establish a Helping Station in Los Angeles where patients might be examined, the status of their cases determined, and such relief given as possible. Dr. F. M. Pottenger was appointed Chief of Staff, and in his absence, Dr. C. C. Browning was made acting Chief of Staff, with Drs. Kress and Thornton as assistants. These men have all given of their time and services freely, and with the aid of a Station Nurse and Visiting Nurse, 246 patients have been cared for up to date. The value of such an institution in the community, therefore, has been unquestionably demonstrated. I may say that no funds of this Association were used in this work, 16 men of Los Angeles having contributed $775.00, of which about $725. has been expended through the Helping Station.

When there shall be a real awakening in the public mind to the vast importance to the community of stopping the fearful waste of life caused by tuberculosis, then our public spirited men and women will come forward, with, at least, as much zeal as does the hog rancher, the cattleman and the orchardist, when cholera, anthrax, or one of those pests which do such damage to the orchards, comes upon him. In either event, neither money nor effort is spared until the infection is rooted out and the property saved from destruction. I will ask you to consider that the average value of a hog on the hoof is only $14.00, and the average value of a steer, or a tree, is from $40. to $50., and that the loss annually by tuberculosis in the human family in the United States of 150,000 persons, at the average age of 35 years, a reliable authority claims is a loss to the community, based upon the net value of a year of human life at the age named, of not less than $240.000.000 per annum. To this, must be added about a million and a quarter cases of tuberculosis existing in the United States all the time; one fourth of these must be supported other than by their own efforts, and where they are heads of families, wives and children dependent upon them must be cared for. The loss of wages alone which should be earned by this one-fourth incapacitated, at the rate of $1.50 per day, figures out $140.000.000 a year. All this to be added to the loss by death, which can not be measured in money value, nor the heartaches, sorrow, misery and distress entailed by this disease, the most insidious and the most difficult of detection of any. Tuberculosis is the great life extinguisher—it destroys more lives, two to one, than all the wars in Christendom. One seventh of all the deaths in the civilized world are caused by tuberculosis. Over 4000 deaths from this disease in California were reported in 1906. Some statistics which I have gathered show that but a very small percentage of these deaths are natives; 10 percent. had lived here less than three months; 25 percent. had lived here less than one year and 60 percent. less than five years. Does this statement suggest to some one that measures should be taken to exclude these bacilli laden people from our State? Let me take a stereoptican and throw on a screen a map of California and have it large enough so there could be shown every house, and over each house that contains a sufferer from this dread scourge, float a cross, who is there in this State brave enough to suggest that each one of those families should be turned back or blotted out? Some of the fairest men and women of this fair land have taken hope and courage for the fight against this dread disease under our sunny skies and amidst our evergreen groves, near the shadow of our mountains; cheeks that were wan have received the color of health, limbs that tottered have become strong, and health, life and joy have taken the place of woe and distress. These men and women have taken their places amongst us in all walks of life, carrying their full burden of responsibility, and contributing of their regained strength to the upbuilding of the Commonwealth. There is, however, some reason for restrictive measures to be taken in connection with the prevalent practice of communities in other States, sometimes by the advice of doctors, and frequently without it, sending to California the indigent invalid, tuberculous or othewise, and treating this State as an organized county farm, or National Charity Hospital. We can not deny them our climate, or our fair fields; but if any community wants to avail itself of these benefits, it should be required to pay for the necessities of life required by those of whose support it has relieved itself, until they shall be able to take care of themselves.

In a book recently published, written by a most profound thinker and a student of Oriental art, a Japanese, a quotation is made from one of the early Chinese writers and philosophers—Soshimon—as follows: "The true beauty of a city lies rather in the happy faces of it's people, than in the towers and ornaments upon it's buildings." If we all agree that this is true, shall we not consider it to be our first and chief duty to co-operate with any agency which has for its primal object, the conservation of the health of every individual in the community.

In such a simple statement may be embodied the aims and purposes of the California Association for the Study and Prevention of Tuberculosis.

